# Early-Onset Retinopathy in Patients With Variants in *SLC6A6* Leading to Impaired Taurine Transport

**DOI:** 10.1001/jamaophthalmol.2025.4875

**Published:** 2025-12-04

**Authors:** Mukhtar Ullah, Atta Ur Rehman, Madhur Shetty, Michael D. Allen, Ehsan Ullah, Sabrina G. Signorini, Cyril Burin des Roziers, Rosalie M. Grijalva, Abdur Rashid, Asad Munir, Alessandra Pia Porretta, Enza Maria Valente, Aime R. Agather, Ioannis Dimopoulos, Robert B. Hufnagel, Edouard Malandain, Juliette Coursimault, Muhammad Ansar, Stylianos E. Antonarakis, Andrea Superti-Furga, Sanaullah Jan, Brian P. Brooks, Giacomo Calzetti, Bin Guan, Mathieu Quinodoz, L. Keith Henry, Carlo Rivolta

**Affiliations:** 1 Institute of Molecular and Clinical Ophthalmology Basel, Basel, Switzerland; 2 Department of Ophthalmology, University of Basel, Basel, Switzerland; 3 Department of Zoology, Faculty of Biological and Health Sciences, Hazara University, Mansehra, Pakistan; 4 Department of Biomedical Sciences, University of North Dakota School of Medicine and Health Sciences, Grand Forks; 5 Ophthalmic Genetics and Visual Function Branch, National Eye Institute, National Institutes of Health, Bethesda, Maryland; 6 Developmental Neuro-Ophthalmology Unit, IRCCS Mondino Foundation, Pavia, Italy; 7 Laboratoire SeqOIA, Paris, France; 8 Service de Médecine Génomique des Maladies de Système et d’Organe, Hôpital Cochin, Paris, France; 9 Department of Ophthalmology, Jules Gonin Eye Hospital-Fondation Asile Des Aveugles and University of Lausanne, Lausanne, Switzerland; 10 Service de cardiologie, Département cœur-vaisseaux, Centre Hospitalier Universitaire Vaudois, Lausanne, Switzerland; 11 CNMR Maladies Cardiaques Héréditaires Rares, APHP, Hôpital Bichat Claude-Bernard, Paris, France; 12 Department of Molecular Medicine, University of Pavia, Pavia, Italy; 13 Neurogenetics Research Center, IRCCS Mondino Foundation, Pavia, Italy; 14 Department of Ophthalmology, Rouen University Hospital and University of Rouen, Rouen, France; 15 Department of Genetics and Reference Center for Developmental Disorders, University of Rouen Normandie, Inserm U1245, CHU Rouen, Rouen, France; 16 Advanced Molecular Genetics and Genomics Disease Research and Treatment Centre, Dow University of Health Sciences, Sindh, Pakistan; 17 Department of Genetic Medicine and Development, University of Geneva, Geneva, Switzerland; 18 Division of Genetic Medicine, University of Lausanne, Lausanne, Switzerland; 19 Department of Ophthalmology, Hayatabad Medical Complex, Peshawar, Khyber Pakhtunkhwa, Pakistan; 20 Pakistan Institute of Community Ophthalmology, Hayatabad Medical Complex, Peshawar, Khyber Pakhtunkhwa, Pakistan; 21 Vista Vision Eye Clinic, Brescia, Italy; 22 Department of Genetics, Genomics and Cancer Sciences, University of Leicester, Leicester, United Kingdom

## Abstract

**Importance:**

Inherited retinal dystrophies are a group of disorders that may lead to progressive vision loss. Improved knowledge of their molecular genetics is important for accurate diagnosis or development of targeted therapies.

**Objective:**

To identify pathogenic variants in the *SLC6A6* gene (encoding TauT, the main transporter for taurine) and assess their role in the molecular pathogenesis of hereditary early-onset retinal dystrophy (EORD) in affected individuals from diverse ethnic backgrounds.

**Design, Setting, and Participants:**

This was a retrospective, multicenter observational study conducted between June 2019 and March 2025, involving 7 affected and 10 unaffected individuals from 4 unrelated families recruited in Pakistan, Italy, the US, and France.

**Exposure:**

Pathogenic variants in *SLC6A6* in individuals with EORD.

**Main Outcomes and Measures:**

Genetic, clinical, and functional outcomes of pathogenic variants in *SLC6A6* in individuals with Leber congenital amaurosis (LCA) and EORD. All patients underwent standard clinical examinations, including visual acuity, full-field electroretinography, and multimodal retinal imaging, followed by measurement of fasting plasma taurine levels. In vitro and ex vivo taurine transport and membrane trafficking assays in human embryonic kidney (HEK)-293 cells, as well as patient-derived fibroblasts, were also performed.

**Results:**

All 7 affected individuals exhibited LCA/EORD, with extraocular findings in some. Genetic analysis identified homozygous pathogenic *SLC6A6* variants in all affected individuals, while unaffected relatives were heterozygous carriers. Families 1 and 2 carried missense variants p.(Thr249Ile) and p.(Ala294Thr), while families 3 and 4 carried truncating variants—a deletion of exon 11 and p.(Thr113Ter), respectively. Functional studies demonstrated that both missense variants are associated with complete loss of taurine transport in HEK-293 cells and patient-derived fibroblasts. Additionally, irrespective of the variants considered, plasma taurine levels in affected individuals were reduced compared with heterozygous carriers (difference between means, −31.7 µmol/L; 95% CI, −42.7 to −20.8; *P* < .001) and healthy control individuals (difference between means, −37.7 µmol/L; 95% CI −41.6 to −33.8; *P* < .001).

**Conclusions and Relevance:**

These findings confirm and expand the role of biallelic variants in *SLC6A6* in association with LCA/EORD due to impaired taurine transport. These findings suggest that patients with a diagnosis of *SLC6A6*-related LCA/EORD may be candidates for investigational oral taurine supplementation.

## Key Points

**Question:**

Do newly identified biallelic *SLC6A6* variants confirm its involvement in retinal degeneration and expand the genetic and clinical spectrum of the disease?

**Findings:**

In this cohort study, 7 affected and 10 unaffected individuals from 4 unrelated families with Leber congenital amaurosis/early-onset retinal dystrophy were investigated. Findings suggested that the observed clinical phenotypes in all the affected individuals were due to biallelic pathogenic variants in the *SLC6A6* gene, encoding the taurine transporter TauT.

**Meaning:**

Impaired TauT function was associated with disrupted taurine transport and early-onset retinal degeneration in this study.

## Teaser

This cohort study evaluates the association between variants in *SLC6A6* and hereditary early-onset retinal dystrophy.

## Introduction

Leber congenital amaurosis (LCA) and early-onset retinal dystrophy (EORD) belong to the large spectrum of inherited retinal diseases (IRDs), characterized by severe visual impairment or blindness in young children.^1,2^ LCA patients present with severe visual impairment at birth or within the first 6 months of life. In contrast, visual loss in EORD individuals is typically detected after early infancy but before age 5 years.^3^ Differentiating LCA from EORD is often difficult because of their substantial clinical overlap and because fundus examination results may appear normal in the early stages of the disease.^4^ At least 22 different genes are known to cause LCA/EORD.^5^

The human solute carrier family 6 member 6 gene (*SLC6A6*) encodes a ubiquitously expressed taurine transporter (TauT) that regulates the intracellular taurine content in many tissues, including brain, retina, heart, kidney, liver, and skeletal muscles.^6,7^ TauT actively shuttles taurine from the extracellular environment into the cell against its concentration gradient in a sodium and chloride ion–dependent manner.^6,7^ The importance of taurine in retinal development, photoreceptors maintenance, and cellular homeostasis was recognized as early as the 1970s when studies involving taurine-deficient cats demonstrated progressive retinal degeneration.^8^ Subsequent research has shown that cellular intake of taurine supports photoreceptors viability, regulates intracellular calcium signaling, and acts as an antioxidant.^9^ Similarly, Slc6a6 knockout mice exhibit multisystem pathologies, including retinal degeneration, cardiomyopathy, skeletal defects, and sensory dysfunctions.^6,10,11,12,13,14^ However, a direct link between *SLC6A6* and human diseases has been established only recently, following the identification of 2 unrelated pedigrees with LCA/EORD and biallelic recessive variants in this gene.^15,16^ Patients from 1 of these 2 families also had cardiomyopathy.^16^ Based on these findings, oral supplementation of taurine has been investigated as a possible therapeutic intervention.^16,17^

Here, we report on 7 patients from 4 families of Pakistani, Italian, Egyptian (living in the US), and Turkish (living in France) origin affected by LCA/EORD. We associate the phenotype in each family with distinct pathogenic variants in *SLC6A6*, detected by whole-exome/genome sequencing (WES/WGS). Furthermore, we used in vitro models to examine how the 2 missense variants affect SLC6A6 protein function.

## Methods

### Recruitment of Patients

All participants, including patients and healthy relatives, were enrolled after their parents or legal guardians were informed about the study purpose and provided written informed consent. No stipends or incentives were provided for participation; however, in some cases, the cost of clinical tests specifically required for the study was covered. This investigation was approved by the institutional review board of the following institutions: Hazara University, the Ethikkommission Nordwest-und Zentralschweiz, the IRCCS Mondino Foundation, and the National Eye Institute. The study adhered to the guidelines established by the Association for Research in Vision and Ophthalmology, as well as the Declaration of Helsinki for the use of human subjects in biomedical research. The French family provided direct written consent for biological material to be used for research. Furthermore, the study was conducted and reported in accordance with the Strengthening the Reporting of Observational Studies in Epidemiology (STROBE) reporting guideline.

### Clinical Examination of Patients

All patients underwent detailed ophthalmological and systemic assessments at their respective clinical centers in Pakistan, Italy, the US, and France. Examinations included visual function testing, multimodal retinal imaging, International Society for Clinical Electrophysiology of Vision standard full-field electroretinogram (ERG), electrocardiogram (ECG), echocardiogram (ECHO), and plasma taurine levels, performed according to local protocols. Detailed clinical procedures and instrumentation are provided in the eMethods in Supplement 1.

### DNA Extraction and Sequencing

WES/WGS was performed on DNA extracted from patients; detailed experimental and computational methods are described in the eMethods in Supplement 1.

### Cell Culture and *SLC6A6* Mutagenesis and Transfection

Functional characterization of missense variants was performed in transiently transfected human embryonic kidney (HEK)-293 cells and patient-derived fibroblasts. ^3^H-taurine uptake and TauT cell surface expression experiments are also described in the eMethods in Supplement 1.

### Statistical Analysis

Plasma taurine, ^3^H-taurine uptake, and TauT surface expression were analyzed using unpaired *t* tests with Welch correction; Holm-Šídák adjustment was applied for multiple comparisons where appropriate. All *P* values were 2-sided. Analyses were performed in Prism version 10.5.0 (GraphPad).

## Results

### Clinical and Demographic Data

**Figure 1.**
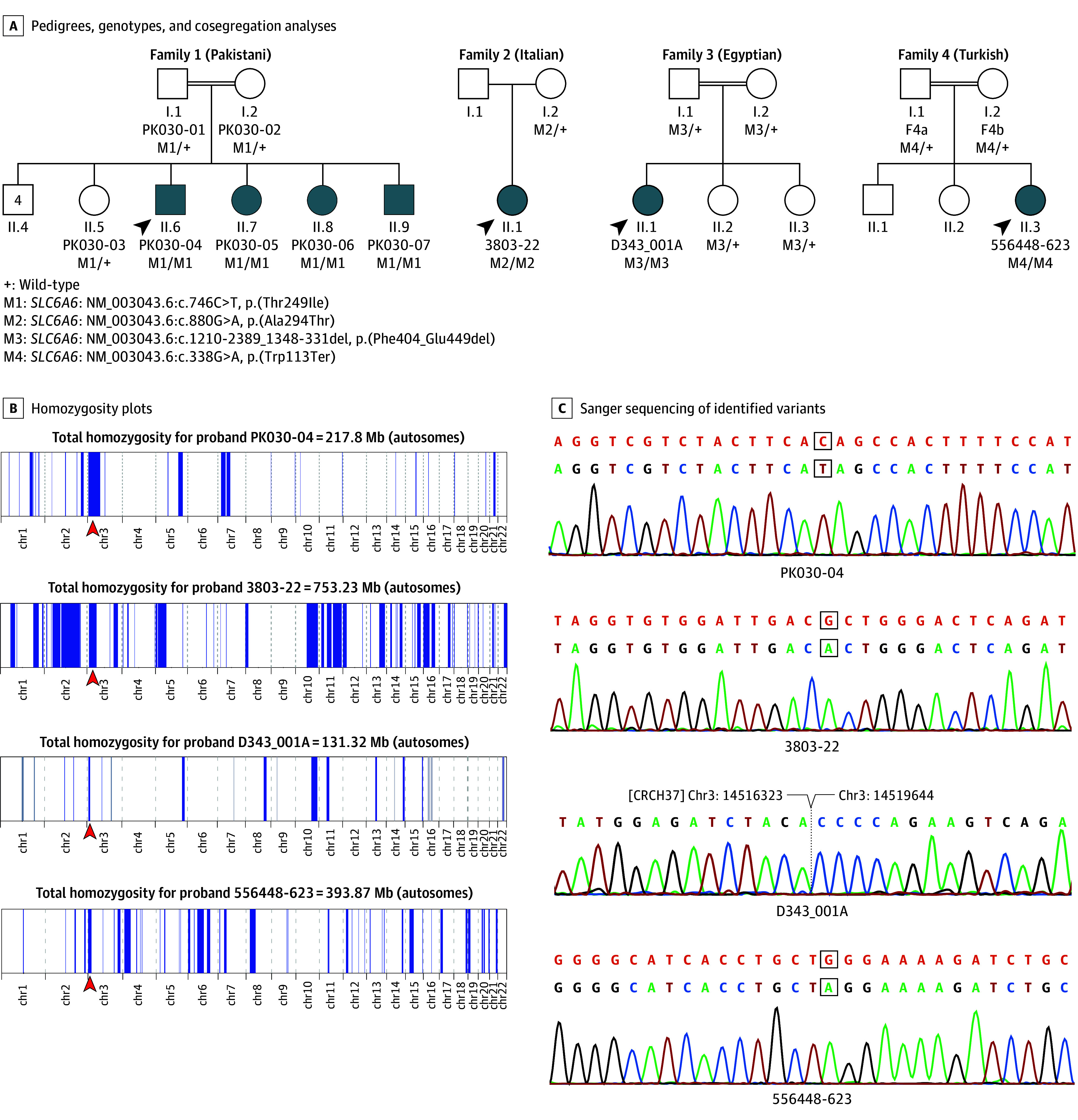
Genetic Analysis of Included Patients and Their Families A, Pedigrees, genotypes, and cosegregation analyses. B, Homozygosity plot of probands PK030-04, 3803-22, D343_001A, and 556448-623. Blue lines indicate runs of homozygosity and the red arrowheads point to the genomic region containing *SLC6A6*. C, Sanger sequencing of the variants identified, including the breakpoint of the deletion detected in D343_001A. The reference sequence of these DNA stretches is shown in the top row in orange.

**Figure 2.**
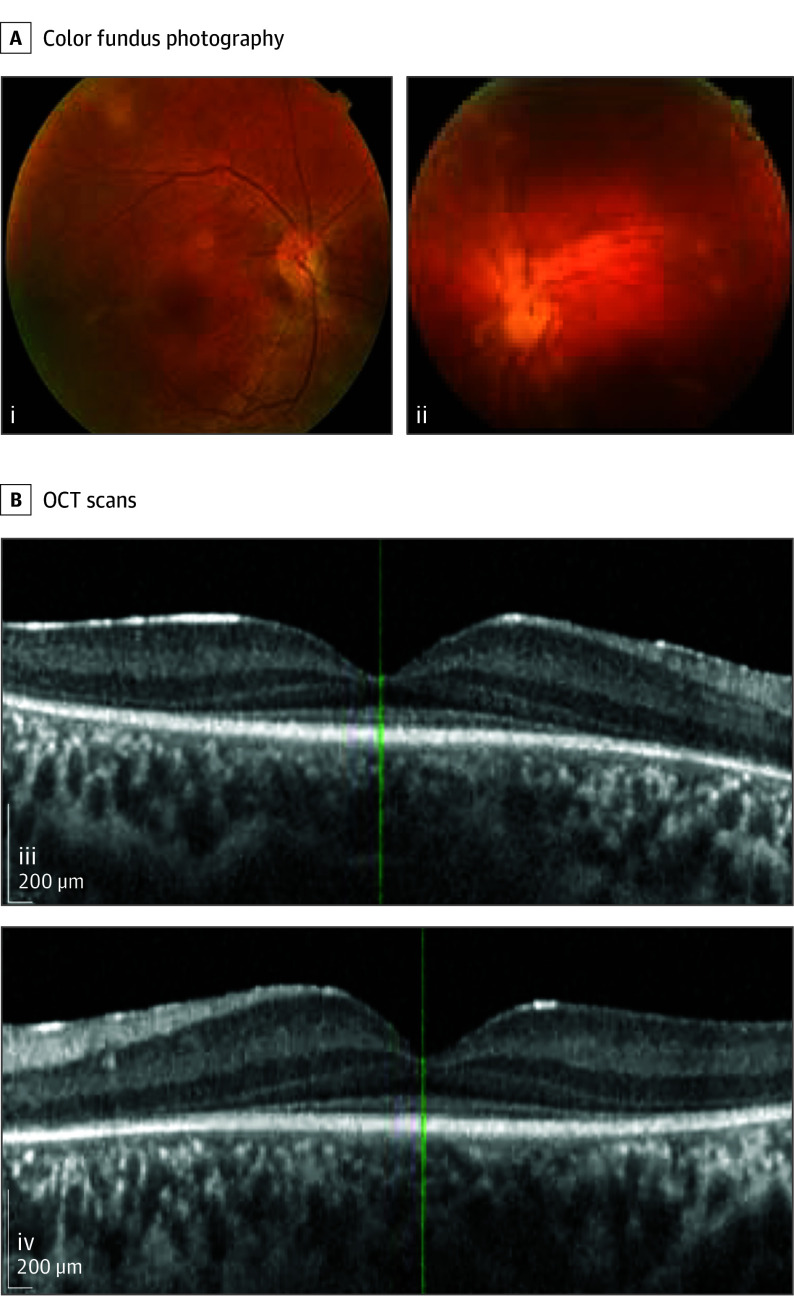
Fundus Appearance and Optical Coherence Tomography (OCT) of *SLC6A6*-Associated Retinopathy A, Color fundus photography of the right (i) and left (ii) eyes of patient PK030-05 (family 1, age 14 years) showing attenuated retinal vasculature and slightly blurred optic disc margins, especially on the nasal side. The yellow blobs are photographic artifacts. B, Macular OCT of the right (iii) and left (iv) eyes of the same individual showing a central island of the outer nuclear layer that abnormally decreases in thickness with eccentricity. External limiting membrane and ellipsoid zone line appear not well delineated and limited to a central island.

Family 1 is from northwestern Pakistan and was composed of 2 unaffected parents, who were first cousins, and their offspring (6 male and 3 female) (Figure 1A). No other affected individuals were known by history. Four of the children had visual impairment and nystagmus since birth, with gradually deteriorating visual function over the years. All 4 patients presented with severely depressed scotopic and photopic ERG recordings in both eyes (eTable 1 in Supplement 1). Based on the presence of poor vision and nystagmus soon after birth and very reduced ERGs, the patients were given a clinical diagnosis of LCA.^18,19,20^ Fundus pictures, ocular ultrasound, and macular optical coherence tomography (OCT) images are shown in Figure 2A and B and eFigure 1 in Supplement 1. Nonocular phenotypes, specifically cardiac abnormalities, were evaluated by ECG and ECHO in all 4 affected individuals. Cardiac structure was normal in all patients; however, short PR intervals were observed in all patients, while positive voltage criteria for left ventricular hypertrophy and mild repolarization abnormalities were noted only in the proband PK030-04 (eTable 1 in Supplement 1).

Family 2 was from Italy and was composed of unaffected parents and an affected female child. Family history did not reveal any other individuals affected by visual impairment or heart disease. The patient was examined when she was 5 years old because of mild to moderate intellectual disability (IQ, 62) associated with oculomotor abnormalities such as alternating fixation, smooth pursuit impairment, and hypometric saccades associated with hypermetropia and normal fundus examination results, within a framework of developmental motor coordination disorder. The ocular findings until the age of 9 years old are summarized in eTable 1 in Supplement 1. The ERG showed a depressed scotopic and photopic response in both eyes. The patient was given a clinical diagnosis of EORD. ECG showed tachycardic sinus rhythm, short PR interval (80 milliseconds), and mild right conduction delay, while transthoracic ECHO results were normal with preserved left ventricular size and function (ejection fraction [EF], 60%) (eTable 1 in Supplement 1).

Family 3, Egyptian by origin, resided in the US and had a history of visual impairment in 1 of their 3 female children. The unaffected parents were first cousins (Figure 1A). The affected proband was examined at age 11 years. The parents reported night blindness onset between age 1 and 2 years, followed by progressive deterioration of daytime and color vision. Initially she was able to track near objects and smile at her parents. Between age 5 and 6 years, intermittent exotropia and nystagmus were noted. The ophthalmological features at presentation (age 11 years) are described in eTable 1 in Supplement 1. ERG responses were extinguished for both scotopic and photopic conditions. Fundus appearance and macular OCT imaging were consistent with advanced retinal degeneration (eFigure 1 in Supplement 1). The patient was given a clinical diagnosis of EORD. At age 18 years, progression of macular atrophy and clumped pigmentary degeneration was noted on widefield imaging compared with her initial presentation (eFigure 1 in Supplement 1). Based on her positive *SLC6A6* molecular testing at this visit, supplementation with oral taurine, 1 g/d, was suggested, increasing to 2 g/d if tolerated. Baseline plasma taurine levels were reduced at 5 nmol/mL. In a follow-up assessment 8 months later, her examination and full-field stimulus test (FST) measurements were essentially unchanged, but plasma taurine levels had increased slightly to 12 nmol/mL. She continued 2 g/d taurine supplementation. When she returned 9 months later, her examination results (including FST) were unchanged. Her plasma taurine measurements, however, were further increased to 29 nmol/mL; now within the age-adjusted normal range of 21 to 123 nmol/mL. Results for routine plasma chemistries, urinalysis, liver function tests, urine organic acids, and serum pyruvate/lactate were normal. Between the first and the last ophthalmological examination, the patient had undergone an ECG that showed normal sinus rhythm with a ventricular rate of 79 bpm and a precordial pattern potentially suggesting right ventricular hypertrophy vs early precordial transition. ECHO results, however, revealed an EF of 56% with no evidence of right or left chamber dilation or hypertrophy.

Family 4 was of Turkish origin and consisted of 2 healthy parents who were first cousins (Figure 1A). The patient was the couple’s third child and was referred to a medical geneticist at the age of 7 months due to facial dysmorphic features, ocular fixation abnormalities, and motor dysfunction. The first ophthalmological examination revealed esotropia and moderate hyperopia. Pendular nystagmus appeared around 12 months of age, with substantial vision loss. The clinical features are detailed in eTable 1 in Supplement 1; fundus appearance and OCT images are shown in eFigure 1 in Supplement 1. At age 4 years, ERG showed severely reduced scotopic and photopic responses, confirming the diagnosis of EORD. At age 7 years, cardiological evaluation showed normocardic sinus rhythm (99 bpm), QTc of 413 milliseconds, and normal ECHO (EF, 65%). Her 2 older siblings, aged 13 and 9 years, were asymptomatic. Plasma taurine levels in all 7 affected individuals were reduced compared with heterozygous carriers and healthy control individuals (eFigure 4 and eTable 1 in Supplement 1).

### Genetic Analysis

**Figure 3.**
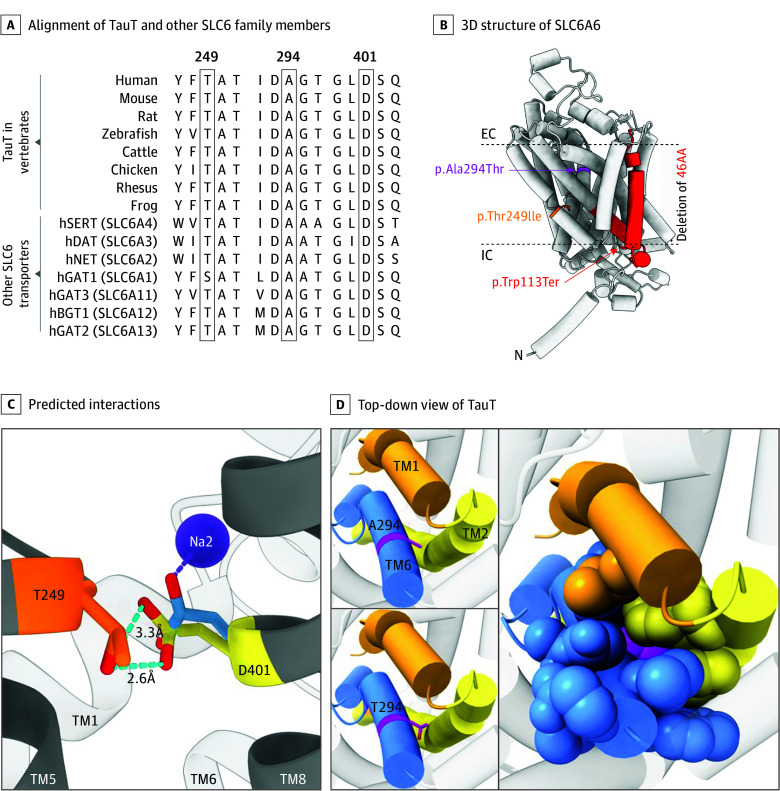
Amino Acid Conservation, AlphaFold Structure Prediction of TauT, and the Impact of Missense Variants A, Alignment of TauT and other SLC6 family members using Jalview^23,24,25^ for residues proximal to Thr249, Ala294, and Asp401 (the coordination partner of Thr249) reveals their highly conserved nature. Residue numbering is based on human TauT. B, Predicted 3-dimensional (3D) structure of SLC6A6 (AlphaFold, AF-P31641-F1-v4). The missense and nonsense variants from families 1 (orange), 2 (magenta), and 4 (red) and the deletion of the eighth α-helix transmembrane domain in family 3 (red) are shown with extracellular (EC) and intracellular (IC) demarking the extra- and intracellular margins, respectively. C, Predicted interactions between Thr249 (orange), Asp401 (yellow), and the Na2 Na^+^ overlaid from human SERT (hSERT) cryo–electron microscopy structure (PDB 7LIA) aligned in ChimeraX using the Matchmaker tool. The coordination of Na^+^ in Na2 of hSERT by D437 (homologous to residue D401 in TauT) is shown in blue. D, Top-down view of TauT with the extracellular side of the protein toward the observer. Residue Ala294 (magenta stick, left upper panel) is located on the C-terminal side of TM 6 (blue), with close interaction between TM 2 (yellow) and TM 1 (orange) residues. Substitution p.(Ala294Thr) introduces additional bulk and polarity (lower left panel) into this densely packed, largely hydrophobic region (right panel, residues within 6Å of Ala294 shown as space filling).

WES of the family 1 proband, PK030-04 (Figure 1A), revealed a homozygous missense variant—NM_003043.6:c.746C>T, p.(Thr249Ile)—in *SLC6A6*. This variant was absent from relevant population databases (gnomAD v.4.1 and Greater Middle East [GME] variome^21,22^) and was located in a large homozygous stretch on chromosome 3 (66.54 Mb) (Figure 1B). Thr249 is located in transmembrane domain (TM) 5 of SLC6A6. This residue is also highly conserved across vertebrates and other members of the human SLC6 family (Figure 3A and B^23,24,25^), suggesting the introduction of the larger, hydrophobic isoleucine would negatively impact TauT structure and function. In silico predictors, including MutScore, further supported the pathogenicity of the variant^26^ (eTable 2 in Supplement 1).

All available members of the family were genotyped by Sanger sequencing. Both parents and other healthy individuals were heterozygous carriers of p.(Thr249Ile), whereas all affected individuals were homozygous (eFigures 1A and 2A in Supplement 1). Since the variant cosegregated with disease in an autosomal recessive pattern, p.(Thr249Ile) was classified as likely pathogenic based on American College of Medical Genetics (ACMG) guidelines,^27^ as described in eTable 2 in Supplement 1.

Similarly, WES analysis of the proband from family 2 revealed a homozygous missense variant—NM_003043.6:c.880G>A, p.(Ala294Thr)—in *SLC6A6*, comprised within a large autozygous region within chromosome 3 (Figure 1A and B). In gnomAD, the variant is reported to be extremely rare (allelic frequency = 5.45 × 10^−5^) and is not seen homozygously. Ala294 in TM 6 is also highly conserved across various vertebrate species and other human SLC6 transporters (Figure 3A, B, and D). The AlphaFold SLC6A6 structure prediction revealed a tightly packed region around Ala294 within a 6Å radius (Figure 3D). The substitution of Ala294 with threonine p.(Ala294Thr) introduces a bulkier and polar amino acid into this hydrophobic environment potentially disrupting side-chain packing and Van der Waals interactions (Figure 3D). This DNA change cosegregated with disease in the family (Figure 1A; eFigure 2B in Supplement 1). According to ACMG guidelines,^27^ the variant was classified as pathogenic (eTable 2 in Supplement 1). Homozygosity mapping on WES data by AutoMap^28^ also showed extensive and unusually high autozygosity (Figure 1B), suggesting the mild intellectual deficit displayed by this patient may be due to unrecognized homozygous defects in other genes, rather than in *SLC6A6*.

Genome sequencing of the proband from family 3 revealed a 3320-bp homozygous deletion—NM_003043.6:c.1210-2389_1348-331del, p.(Phe404_Glu449del)—which encompassed the entire exon 11 of the *SLC6A6* gene, encoding 46 aa residues belonging to TM 8 of SLC6A6 (Figure 3B). This deletion has not been previously observed in the gnomAD or Decipher databases,^29^ indicating that it is a very rare event. The presence of this rearrangement was validated by sequencing the resulting breakpoint by the Sanger technique (Figure 1C) and confirmed to cosegregate with disease in the pedigree as an autosomal recessive deleterious variant (Figure 1A; eFigure 2C in Supplement 1). According to the ACMG guidelines,^27^ this deletion can be classified as likely pathogenic (eTable 2 in Supplement 1).

In family 4, trio genome sequencing identified the cause of the proband’s severe retinopathy by detecting a homozygous pathogenic truncating variant—NM_003043.6: c.338G>A, p.(Trp113Ter)—in the *SLC6A6* gene, with parents being heterozygous carriers (Figure 1A; eFigure 2D in Supplement 1). This variant, which creates a premature stop codon in exon 4 and likely triggers nonsense-mediated decay, has not been observed in the gnomAD v4.1. Taken together, the variant was classified as likely pathogenic based on ACMG criteria (eTable 2 in Supplement 1).

### Functional Validation of the Missense Variants

**Figure 4.**
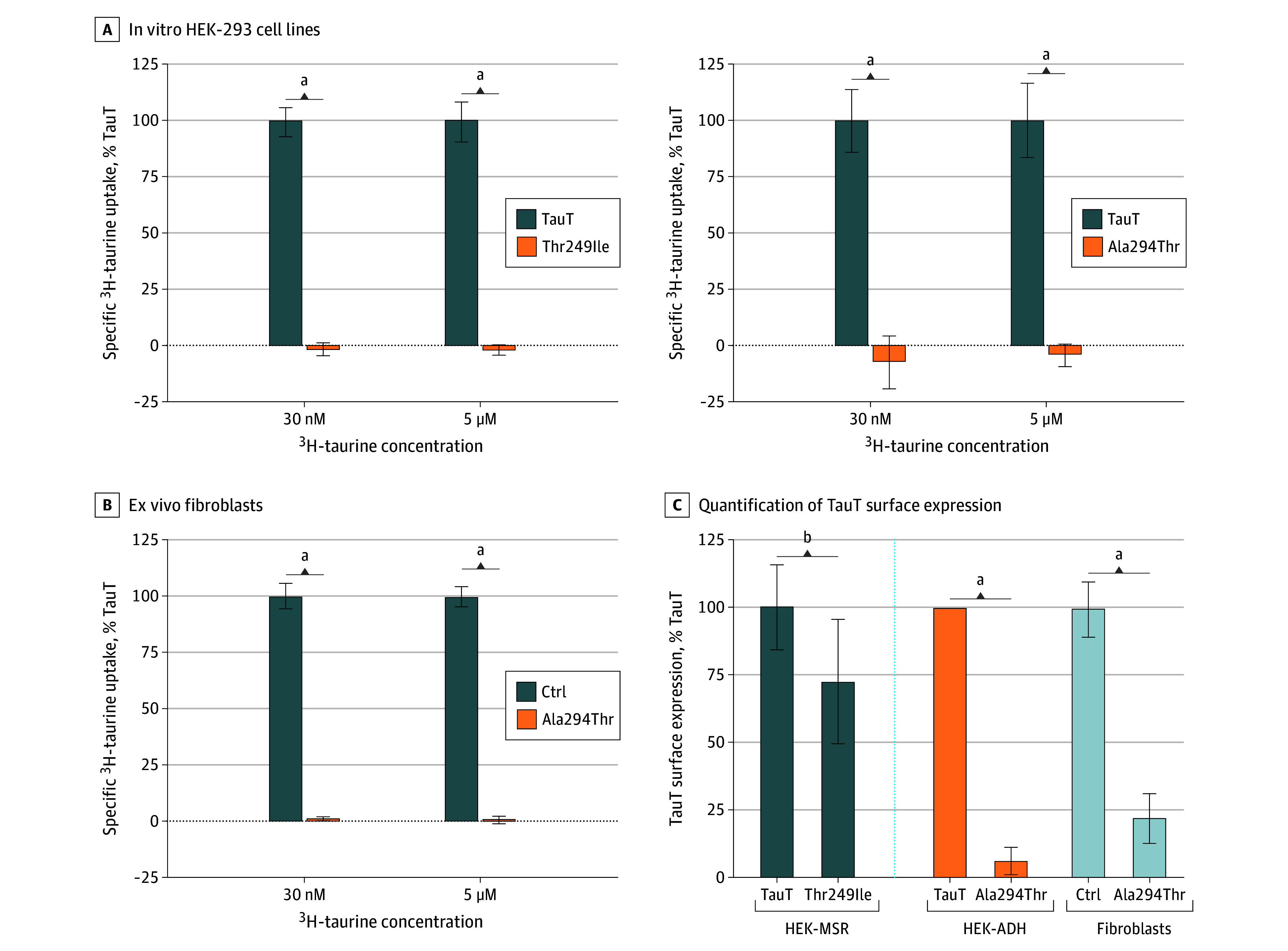
Reduced Taurine Uptake and Surface Expression in Cells Expressing the p.(Thr249Ile) and p.(Ala294Thr) Variants A, ^3^H-taurine uptake in human embryonic kidney (HEK)-293 cells expressing exogenous wild-type TauT compared with the p.(Thr249Ile) variant (left; n = 8) and the p.(Ala294Thr) variant (right; n = 6). The contribution of endogenous TauT was subtracted and the values were normalized to percent wild-type uptake. Expression of nonfunctional variant TauT appeared to negatively impact endogenous TauT activity or expression, resulting in negative values. B, ^3^H-taurine transport in patient-derived fibroblasts homozygous for p.(Ala294Thr) revealed a near complete loss of taurine transport compared with control fibroblasts (n = 6). All uptake assays were performed at low and high concentrations of taurine (30 nM and 5 μM). C, Quantification of TauT surface expression in HEK-293 cell lines and fibroblasts. HEK cell lines: p.(Thr249Ile), n = 6 and p.(Ala294Thr), n = 4; fibroblasts: p.(Ala294Thr), n = 3. For representative blots see eFigure 3A-C in Supplement 1. All n values are independent experiments presented as mean (SD). ^a^*P* < .001. ^b^*P* < .01.

Our functional validation study revealed that the p.(Thr249Ile) variant displayed a complete loss of function compared with wildtype TauT in single-point radioactive taurine uptake assays, at both low (30 nM) and high (5 μM) taurine concentrations (Figure 4A). To determine if functional loss was due to a plasma membrane trafficking defect, we performed surface biotinylation analysis to tag, purify, and quantify TauT at the plasma membrane and found that surface levels of the p.(Thr249Ile) variant were only 72% of wildtype (95% CI, −47.25 to −8.22; *P* = .009), indicating diminished surface trafficking and that the loss of function likely resulted from kinetic and surface expression deficiencies (Figure 4C).

Similarly, the p.(Ala294Thr) variant was amorphic, with taurine uptake slightly below baseline even at 5 μM taurine after subtracting endogenous TauT function in HEK-ADH cells (Figure 4A), making decreased substrate affinity an unlikely contributor. Surface biotinylation studies revealed a 94% reduction of p.(Ala294Thr) compared with wildtype (95% CI, −89.54 to −67.39; *P* < .001) (Figure 4C), indicating that most of the p.(Ala294Thr) variant misfolds and fails to traffic to the plasma membrane and that the remaining 6% reaching the surface lacks functional transport. These findings were corroborated using patient-derived homozygous p.(Ala294Thr) fibroblasts which exhibited a 99% reduction in transport (Figure 4B) and a 76% (95% CI, −102.1 to −86.21; *P* < .001) decrease in surface expression relative to control fibroblasts (Figure 4C).

## Discussion

This cohort study reaffirms and strengthens the involvement of *SLC6A6* in LCA/EORD and expands its genetic and functional spectrum by identifying pathogenic variants that impair taurine transport. Four families with SLC6A6-associated LCA/EORD were identified whose ocular phenotype and low plasma taurine levels aligned with recent findings and are in part reminiscent of the phenotypes observed in TauT knockout mice.^6,12,13,15,16^

The first pedigree from Pakistan carried the p.(Thr249Ile) missense, which had not been previously reported either in association with disease or in the context of normal human genetic variation. In vitro studies in HEK-293 cells expressing the p.(Thr249Ile) variant showed a complete loss of taurine transport attributed to both a substantial reduction in cell surface trafficking and the absence of transport activity among the limited number of transporters that reached the cell surface. An AlphaFold predicted structure of TauT indicates that Thr249, located on TM 5, interacts with TM 8 residue Asp401^30,31^ (Figure 3C). Both residues are highly conserved among TauT proteins across species and within the SLC6 family (Figure 3A). Notably, the homologous residue to Thr249 in the dopamine transporter (SLC6A3), Thr269, is predicted to coordinate the Na^+^ ion released from the Na2 site during the transition to the inward-facing conformation^32^ and may participate in the hinge motion associated with translocation.^33^ Furthermore, mutational data on the homologous residue, Thr284, in the serotonin transporter (SERT, SLC6A4), shows a functional impact on transport kinetics^34^ and cryo-EM structures reveal that SERT residue Thr284 along with Asp437 (homologous to TauT Asp401) are part of the conformationally dynamic domain attributed to coordinating sodium ions during translocation^35^ (Figure 3C). In addition, SNAP2, an in silico tool that uses neural networks to predict the functional impact of amino acid substitutions in proteins, yielded a score of 92 for p.(Thr249Ile) (range, −100 [lowest impact] to 100 [highest impact]), suggesting that this substitution deleteriously affects TauT function.^36^ Collectively, these findings support a role for TauT Thr249 in transport structure and function through the coordination of Asp401 and Na^+^ ions during the translocation process.

The p.(Ala294Thr) missense variant, identified in homozygosity in a patient from family 2, also results in an SLC6A6 amorph with a high likelihood of misfolding based on the decrease in plasma membrane expression and a SNAP2 score of 38. This finding is consistent with Ala294 being a highly conserved residue located at the top of TM 6, which forms a tightly packed domain with residues from TM 1 and TM 2 (Figure 3D). The extra bulk and polarity added by the threonine substitution likely interfere with proper folding causing loss of function, especially given that TM 1 and TM 6 are known to be dynamic helices critical to substrate recognition and translocation in other SLC6 proteins.^35,37,38,39^

The defects in taurine transport resulting from the large genomic deletion and nonsense DNA change detected in the patients from families 3 and 4 were not modeled in silico since both genetic variants are unlikely to yield a functional transporter. Notably, the gnomAD database reports multiple control individuals with heterozygous loss-of-function variants (such as these patients’ parents) but no homozygotes, suggesting that TauT does not exhibit haploinsufficiency and that the disease state occurs when loss of activity exceeds 50%. Moreover, results with the variant proteins presented here corroborate previous observations in patient-derived fibroblasts and mononuclear cells from other *SLC6A6* variants.^15,16^

The ocular phenotype common to the 7 patients was LCA/EORD, highlighting the importance of SLC6A6 for photoreceptor survival early in life. In contrast to our previous study on TauT variants,^16^ no clear pathological cardiac phenotype has been identified as a shared feature among patients. A single proband (PK030-04) had abnormal ECG results, suggesting a potential left ventricular hypertrophy that was not supported by ECHO. Therefore, our data confirm an association between *SLC6A6* and LCA/EORD but not heart defects. However, since cardiac work-up was performed in childhood or in early adolescence in almost all patients, we cannot exclude the presence of age-dependent latent or evolving phenotypes affecting the heart. For these reasons, and considering the available preclinical findings, a cardiological follow-up may be advisable for such patients. Lack of clinical effects on other organs and tissues in these patients may suggest functional redundancy by the presence of alternative cell- and tissue-specific taurine transporters. For instance, Zhou et al^40^ reported that the *SLC6A13* gene, encoding the GABA transporter 2, is predominantly associated with taurine transport in hepatocytes and contributes to 50% of the taurine content in the liver.

### Limitations

This study has 2 main limitations. First, the assessment of systemic manifestations was not uniform across all affected individuals, which may have limited the detection of subtle extraocular features. Second, functional studies were performed in heterologous cell systems and patient-derived fibroblasts, which may not fully recapitulate the complex retinal microenvironment. Despite these limitations, the consistent genotype-phenotype correlation and functional findings across unrelated families provide robust evidence supporting the pathogenicity of the detected *SLC6A6* variants.

## Conclusions

In conclusion, this study investigated 4 unrelated families with autosomal recessive LCA/EORD associated with biallelic pathogenic variants in the *SLC6A6* gene, extending observations on single pedigrees from previous reports^15,16,17^ and confirming the association between SLC6A6/TauT and retinal disease. Our findings indicate that the observed clinical phenotype is the likely result of the disruption of taurine transport, thus suggesting oral taurine supplementation may be a viable investigational therapy for patients with *SLC6A6*-related disease.

## Notes

Supplement 1. eMethods eResults eReferences eFigure 1. Fundus appearance of SLC6A6-associated retinopathy eFigure 2. Additional data on genotyping and co-segregation analyses eFigure 3. Surface biotinylation immunoblots from HEK-MSR and fibroblasts eFigure 4. Plasma taurine levels in patients and controls eTable 1. Demographic and clinical characteristics of patients eTable 2. Results from in silico tools assessing all variants reported in this study

Supplement 2. Data Sharing Statement
