# Supplementary material for: Early-Onset Retinopathy in Patients With Variants in SLC6A6 Leading to Impaired Taurine Transport
Source: JAMA Ophthalmol. 2025 Dec 4;144(1):70–8. doi: 10.1001/jamaophthalmol.2025.4875 (PMC12679424; doi:10.1001/jamaophthalmol.2025.4875)
Supplement: Supplement 1. — eMethods eResults eReferences eFigure 1. Fundus appearance of SLC6A6-associated retinopathy eFigure 2. Additional data on genotyping and co-segregation analyses eFigure 3. Surface biotinylation immunoblots from HEK-MSR and fibroblasts eFigure 4. Plasma taurine levels in patients and controls eTable 1. Demographic and clinical characteristics of patients eTable 2. Results from in silico tools assessing all variants reported in this study [file jamaophthalmol-e254875-s001.pdf]

# Supplemental Online Content

Ullah M, Ur Rehman A, Shetty M, et al. Early-onset retinopathy in patients with variants in *SLC6A6* leading to impaired taurine transport. *JAMA Ophthalmol*. Published online December 4, 2025.
doi:10.1001/jamaophthalmol.2025.4875

**eMethods**
**eResults**
**eReferences**
**eFigure 1.** Fundus appearance of SLC6A6-associated retinopathy
**eFigure 2.** Additional data on genotyping and co-segregation analyses
**eFigure 3.** Surface biotinylation immunoblots from HEK-MSR and fibroblasts
**eFigure 4.** Plasma taurine levels in patients and controls
**eTable 1.** Demographic and clinical characteristics of patients
**eTable 2.** Results from in silico tools assessing all variants reported in this study

This supplemental material has been provided by the authors to give readers additional information about their work.

© 2025 Ullah M et al. *JAMA Ophthalmology*.

## eMETHODS

### Recruitment of patients

All participants including patients and healthy relatives were enrolled after their parents or legal guardians were informed about the study purpose and provided written consents. No stipends or incentives were provided for participation; however, in some cases, the cost of clinical tests specifically required for the study was covered. This investigation was approved by the Institutional Review Board (IRB) of all the following institutions: Hazara University (approval code: F.No:185/HU/Zool/2018/583), the Ethikkommission Nordwest- und Zentralschweiz (2019-01660), the IRCCS Mondino Foundation (p-20200051830), the NIH/NEI (NCT01778543 and NCT02077894), and adhered to the guidelines established by the Association for Research in Vision and Ophthalmology (ARVO), as well as the Declaration of Helsinki for the use of human subjects in biomedical research. The French family provided direct written consent for biological material to be used for research. Furthermore, the study was conducted and reported in accordance with the STROBE (Strengthening the Reporting of Observational Studies in Epidemiology) guidelines.

### Clinical examination

All patients from Family 1 underwent a comprehensive ophthalmological examination at two medical facilities: the Department of Ophthalmology at Hayatabad Medical Complex, Peshawar, Pakistan, and the Armed Forces Institute of Ophthalmology, Rawalpindi, Pakistan. Diagnostic tests included best-corrected visual acuity (BCVA) test, color fundus photography, optical coherence tomography (OCT), using the Heidelberg Spectralis (Heidelberg Engineering, Heidelberg, Germany), A- and B-scan ultrasonography using the Aviso system, (Quantel Medical, Clermont-Ferrand, France), and ISCEV standard full-field electroretinography (ERG) using an Espion system (Diagnosys LLC, Lowell, MA, USA).

For extraocular examinations, an electrocardiogram (ECG) and echocardiogram (ECHO) were conducted at a private clinic in Pakistan. Plasma taurine quantification was performed using the cation exchange method on HPLC at the Aga Khan University Hospital Karachi, Pakistan, following an 8 to 10-h fasting period.

The patient from Family 2 underwent a complete neuro-ophthalmological examination at the Center of Child Neuro-Ophthalmology, IRCCS Mondino Foundation in Pavia, Italy, which included fundus examination, the assessment of BCVA, color vision and contrast sensitivity tests, ISCEV standard full-field ERG performed with a Retimax Plus device (CSO, Scandicci, Italy), visual evoked potentials, and OCT using the CIRRUS system (Carl Zeiss Meditec, Dublin, CA, USA). The patient also underwent a cardiological assessment including ECG and ECHO. Plasma taurine quantification was performed using the ion-exchange chromatography method, as part of a metabolic evaluation that included the measurement of plasma and urinary amino acids, as well as urinary organic acids.

The proband from family 3 underwent a comprehensive ophthalmological examination at the National Eye Institute (National Institutes of Health, Bethesda, MD, USA) which included BCVA test, Goldmann visual field, intraocular pressure (IOP) measurement, color fundus photography and mid-wavelength fundus autofluorescence imaging using the Optos ultra-widefield fundus camera (Optos, Marlborough, MA, USA), OCT using the CIRRUS system (Carl Zeiss Meditec, Dublin, CA, USA), full-field stimulus test (FST), and ISCEV standard full-field ERG with an LKC machine (LKC Technologies, Gaithersburg, MD, USA). Systemic testing included routine blood chemistries: liver function tests, urinalysis, urine organic acids, plasma pyruvate, lactate, and amino acids, ECHO, ECG, and audiology evaluations. The two siblings in family 3 had BCVA, OCT (Carl Zeiss Meditec, Dublin, CA, USA), color testing via the Farnsworth D15, and ISCEV standard full-field ERG testing.

The patient from family 4 was examined at the Rouen University Hospital, France, at the Departments of Ophthalmology, Genetics, and Cardiology. She underwent a full ophthalmological examination, multimodal imaging including color fundus photography, fundus autofluorescence imaging, and OCT using the CIRRUS system (Carl Zeiss Meditec, Dublin, CA, USA), and ISCEV standard full-field ERG using a MonPackONE system (Metrovision, Pérenchies, France). From a cardiological perspective, she received an ECG and a transthoracic echocardiogram. Fasting taurine levels were measured under standard conditions for plasma amino acid quantification.

**DNA extraction and sequencing**

The exome data of the proband of Family 1 was already available; his DNA was extracted and sequenced as described previously[1].

In Family 2, DNA was extracted from peripheral blood using an automated extractor (Maxwell). For whole exome sequencing (WES), libraries were prepared using Twist Human Core Kit (Twist Bioscience) and run on a NovaSeq6000 sequencer (Illumina).

Bioinformatic analysis and variant annotation was carried out as previously reported[2]. In brief, variants were retained and prioritized based on predicted effect on protein (coding and splicing variants), minor allele frequency (MAF<1%), coverage (>15X), *in silico* predictions of pathogenicity and segregation pattern. Variants were classified according to the ACMG/AMP criteria[3]. Confirmation of the variant and segregation in the mother was performed by Sanger sequencing and Big Dye Terminator chemistry (Applied Biosystems).

DNA from Family 3 was extracted according to the standard protocol described by Garnai et al.[4]. Short-read genome sequencing on the patient, their parents and siblings were done using short-read genome sequencing generated by an Illumina NovaSeq sequencer, as described previously[5]. Sequencing data were analyzed using a custom bioinformatic pipeline, accessible through the following GitHub links (https://github.com/NIH-NEI/NGS_genotype_calling and https://github.com/NIH-NEI/variant_prioritization). Copy number variants (CNVs) were called using Manta[6] and annotated using AnnotSV[7].

For Family 4, WGS was performed on the patient and her parents at the genomic platform SeqOIA in Paris, France. The library was prepared using the NEB Next Ultra II End repair/A-tailing DNA Library Prep Kit (New England Biolabs) and sequenced in paired ends (2 × 150 bp) using an Illumina Novaseq6000 platform. The reads were aligned to the reference human genome using the BWA-MEM 0.7.15 software package. The GATK haplotype caller (v4.1.7.0; Broad Institute)[8] was used to call SNVs, and ClinSV[9] was used for CNV detection. The resulting variants were then annotated with AnnotSVv2.5.1 in an in-house developed workflow (SeqOIA-IT platform). An assessment of variants' pathogenicity was performed according to the American College of Medical Genetics (ACMG) and the Association for Molecular Pathology (AMP) guidelines.

**Cell culturing, generation of TauT/SLC6A6 mutants, and transient transfections**

Human embryonic kidney (HEK-293) cell lines, GripTite™ 293 MSR (HEK-MSR) (ThermoFisher Scientific) and HEK-ADH as well as patient-derived fibroblast were maintained in a humidified atmosphere of 5% $CO_2$ at 37°C in Dulbecco's Modified Eagle Medium (DMEM) (Cytiva, Wilmington,

DE, USA or Genesee Scientific, El Cajon, CA, USA) supplemented with 5% (HEK-293) or 10% (fibroblast) fetal bovine serum (FBS) (R&D Systems, Minneapolis, MN, USA or Genesee Scientific) and 1% penicillin, streptomycin, and amphotericin B (Sigma-Aldrich, St. Louis, MO, USA). HEK-MSR and HEK-ADH cells were grown under selection with G418 (600 µg/mL) (TOKU-E, Bellingham, WA, USA) and Hygromycin B (20 µg/mL) (TCI America, Portland, OR, USA), respectively. TauT mutants were made using mutagenic primers to generate Thr249Ile (Forward: cgtctacttcaTagccacttttcc; Reverse: accttcccagtggacctg) and Ala294Thr (Forward gaatatctgagtcccagTgtcaatccacacctgtg; Reverse: cacaggtgtggattgacActgggactcagatattc) designed with web-based NEBaseChanger and Agilent QuickChange Primer Design tools, respectively. Mutations were introduced into the pCMV6-SLC6A6 plasmid (RC219698, Origene, Rockville, MD, USA) using the Q5 Site-Directed Mutagenesis (New England Biolabs, Ipswich, MA, USA) and QuickChange Lightning (Agilent, Santa Clara, CA, USA) kits according to manufacturer instructions. Site-directed mutants were verified by Sanger sequencing (Eton Biosciences, San Diego, CA) and whole plasmid sequencing (Plasmidsaurus, Arcadia, CA, USA or Eurofins Genomics, Louisville, KY, USA). For transient transfections, HEK-293 cell lines were plated at a density of 35,000 cells per well in Olympus white, clear bottom 96 well plates (Genesee Scientific). After 24 h, cells were transfected using TransIT-LT1 (Mirus Bio, Madison, WI, USA) according to manufacturer's instructions with optimized ratios for 96 well plates (50ng plasmid DNA, 9 µL serum free media and 0.3 µL TransIT-LT1 per well). Of note, the plasmid DNA for these experiments was isolated on two separate occasions from two independent plasmid extraction procedures to eliminate DNA purification artifacts.

### [$^3$H] Taurine uptake assay

TauT-mediated taurine uptake was assessed using [$^3$H] taurine (tritiated taurine), which enables quantification of intracellular uptake by measuring incorporated radioactivity in cell lysates. [$^3$H] taurine uptake assays were performed 24-48 h after transfection as described previously[10], adapted to 96 well format. Briefly, cells were washed with KRH buffer (130 mM NaCl, 1.3 mM KCl, 2.2 mM $CaCl_2$, 1.2 mM $MgSO_4$, 10 mM HEPES, and 10 mM glucose, pH 7.4), followed by addition of 30nM [$^3$H] taurine or 5 µM taurine (2% [$^3$H] taurine) (Revvity, Waltham, MA, USA or American Radiolabeled Chemicals, Saint Louis, MO, USA) in KRH buffer pH 5.5 and incubated at 37˚C for 10 min (HEK-293 cell lines) or 45 min (fibroblast). Uptake was terminated with 2-3 washes of ice-cold KRH buffer (pH 7.4) or room temperature sodium-free KRH buffer (pH 7.4, 131.3 mM KCl). Cells were solubilized in

Microscint-PS (Revvity) for at least 30 minutes before measurements were taken on a MicroBeta$^2$ microplate counter (Revvity). Non-specific [$^3$H] taurine binding was determined by addition of excess non-labeled taurine (10 mM) (Acros Organics, Thermo Scientific Chemicals, Waltham, MA, USA) 5 min prior to introduction of [$^3$H] taurine. Endogenous taurine uptake was quantified by uptake in parental cell lines transfected using pEGFP-N1 plasmid DNA and subtracted from total uptake values.

**Surface biotinylation and immunoblotting**

For cell surface biotinylation, plating and transfection were carried out as described above, except cells were plated (200,000 cells/well) in standard 24-well tissue culture plates coated in poly-d-lysine or in 24 well Cell+ plates (Sarstedt AG & co. KG, Nümbrecht, Germany) with transfection solution ratios of 200ng DNA, 50µL SFM, 0.6µL TransIT-LT1. All surface biotinylation steps were performed on ice and with ice-cold reagents to prevent protein trafficking and degradation. Cells were washed 2X with phosphate-buffered saline buffer with $Ca^{2+}$ and $Mg^{2+}$ (PBS/CM buffer) (137 mM NaCl, 2.7 mM KCl, 10.1 mM $Na_2HPO_4$, 1.8 mM $KH_2PO_4$, 0.1 mM $CaCl_2$, 1.0 mM $MgCl_2$, and 10 mM glucose, pH 7.4) between each of the biotinylation, quenching, and lysing steps. Biotinylation of surface proteins was initiated by addition of freshly solubilized Sulfo-NHS-SS-biotin (0.5 mg/mL) (Proteochem, Hurricane, UT, USA) and gentle rocking for 25 min. This step was repeated once to ensure complete labeling of external proteins. Two 20 min washes with 100 mM glycine (Sigma-Aldrich) were performed with gentle rocking to quench the NHS crosslinking reaction. Cells were washed 2X with PBS/CM buffer and solubilized with 250 µL RIPA buffer (150 mM NaCl, 5 mM EDTA, pH 8.0, 50 mM Tris, pH 8.0, 1% NP-40, 0.5% sodium deoxycholate, 0.1% SDS) containing Pierce Protease Inhibitor cocktail (ThermoFisher Scientific) and 1mM phenylmethylsulfonyl fluoride (PMSF) for 15 min with gentle rocking. Cell lysates were pooled from 6 wells and centrifuged for 20 min at 4˚C at 16,000 x g followed by collection of the supernatant. Protein was quantified using a BCA reaction, and 50 µg protein was added to a 50% slurry of NeutrAvidin™ Agarose Resin (Thermo Scientific, Rockford, IL, USA) and gently rotated overnight at 4˚C. Resin was pelleted gently at 300 x g for 2 min and washed 3X with RIPA buffer. Resin was incubated with Laemmli buffer containing beta-mercaptoethanol for 30 min at room temperature to elute the protein from the resin. The resin was pelleted at 5,000 x g for 2 min and the eluted protein was subjected to SDS-PAGE and immunoblotted for TauT. Total and surface protein samples were electrophoretically resolved using NOVEX™ 4-20% Tris-Glycine Plus WedgeWell™ gels, along with PageRuler Plus protein (Thermo Fisher Scientific) or ColorBurst™

Electrophoresis Markers (Sigma-Aldrich) ladders. The resolved proteins were transferred to 0.45 µM PVDF membranes and blocked for 2 h at room temperature or overnight at 4˚C with 3-5% bovine serum albumin (BSA) (fraction V) in PBS, followed by incubation with anti-TauT primary antibody (PA5-37460, 1:10000, rabbit polyclonal) (ThermoFisher Scientific) overnight at 4˚C. Membranes were incubated with alkaline phosphatase-conjugated (AP) goat anti-rabbit (A3687, Sigma-Aldrich) for 1 h at room temperature. Membranes were imaged using Immun-Star AP Substrate (Bio-Rad, Hercules, CA, USA) on an Azure c600 (Azure Biosystems, Dublin, CA, USA). Band densitometry analysis was performed using ImageJ[11].

All kinetic and expression analyses were performed using Graphpad Prism Version 10.

## eRESULTS

### Plasma taurine levels in patients and controls

On the basis of the genetic and functional findings mentioned above and the data reported previously in patients with similar genotypes[10,12], we measured fasting taurine levels in the plasma of all patients (n=7) and of five heterozygous carriers from Families 1 and 4. All affected individuals had reduced taurine levels (1 to 10 µmol/L), both with respect to the normal concentration range (36 to 52 µmol/L)[13] and the values displayed by heterozygous carriers (eTable 1 and eFigure 4 Supplement 1). These findings suggest that defects in *SLC6A6* are associated with reduced plasma taurine levels, which in turn may contribute to the development of severe retinal dystrophy.

## eREFERENCES

**1.** Ullah M, Rehman AU, Quinodoz M, et al. A comprehensive genetic landscape of inherited retinal diseases in a large Pakistani cohort. *NPJ Genom Med*. 2025;In press

**2.** D'Abrusco F, Arrigoni F, Serpieri V, et al. Get Your Molar Tooth Right: Joubert Syndrome Misdiagnosis Unmasked by Whole-Exome Sequencing. *Cerebellum*. Dec 2022;21(6):1144-1150. doi:10.1007/s12311-021-01350-8

**3.** Richards S, Aziz N, Bale S, et al. Standards and guidelines for the interpretation of sequence variants: a joint consensus recommendation of the American College of Medical Genetics and Genomics and the Association for Molecular Pathology. *Genet Med*. May 2015;17(5):405-24. doi:10.1038/gim.2015.30

**4.** Garnai SJ, Brinkmeier ML, Emery B, et al. Variants in myelin regulatory factor (MYRF) cause autosomal dominant and syndromic nanophthalmos in humans and retinal degeneration in mice. *PLoS Genet*. May 2019;15(5):e1008130. doi:10.1371/journal.pgen.1008130

**5.** Prasov L, Guan B, Ullah E, et al. Novel TMEM98, MFRP, PRSS56 variants in a large United States high hyperopia and nanophthalmos cohort. *Sci Rep*. Nov 17 2020;10(1):19986. doi:10.1038/s41598-020-76725-8

**6.** Chen X, Schulz-Trieglaff O, Shaw R, et al. Manta: rapid detection of structural variants and indels for germline and cancer sequencing applications. *Bioinformatics*. Apr 15 2016;32(8):1220-2. doi:10.1093/bioinformatics/btv710

**7.** Geoffroy V, Herenger Y, Kress A, et al. AnnotSV: an integrated tool for structural variations annotation. *Bioinformatics*. Oct 15 2018;34(20):3572-3574. doi:10.1093/bioinformatics/bty304

**8.** DePristo MA, Banks E, Poplin R, et al. A framework for variation discovery and genotyping using next-generation DNA sequencing data. *Nat Genet*. May 2011;43(5):491-8. doi:10.1038/ng.806

**9.** Minoche AE, Lundie B, Peters GB, et al. ClinSV: clinical grade structural and copy number variant detection from whole genome sequencing data. *Genome Med*. Feb 25 2021;13(1):32. doi:10.1186/s13073-021-00841-x

**10.** Ansar M, Ranza E, Shetty M, et al. Taurine treatment of retinal degeneration and cardiomyopathy in a consanguineous family with SLC6A6 taurine transporter deficiency. *Human molecular genetics*. 2020;29(4):618-623.

**11.** Schneider CA, Rasband WS, Eliceiri KW. NIH Image to ImageJ: 25 years of image analysis. *Nat Methods*. Jul 2012;9(7):671-5. doi:10.1038/nmeth.2089

**12.** Preising MN, Görg B, Friedburg C, et al. Biallelic mutation of human SLC6A6 encoding the taurine transporter TAUT is linked to early retinal degeneration. *The FASEB journal*. 2019;33(10):11507-11527.

**13.** Trautwein EA, Hayes KC. Taurine concentrations in plasma and whole blood in humans: estimation of error from intra- and interindividual variation and sampling technique. *Am J Clin Nutr*. Oct 1990;52(4):758-64. doi:10.1093/ajcn/52.4.758

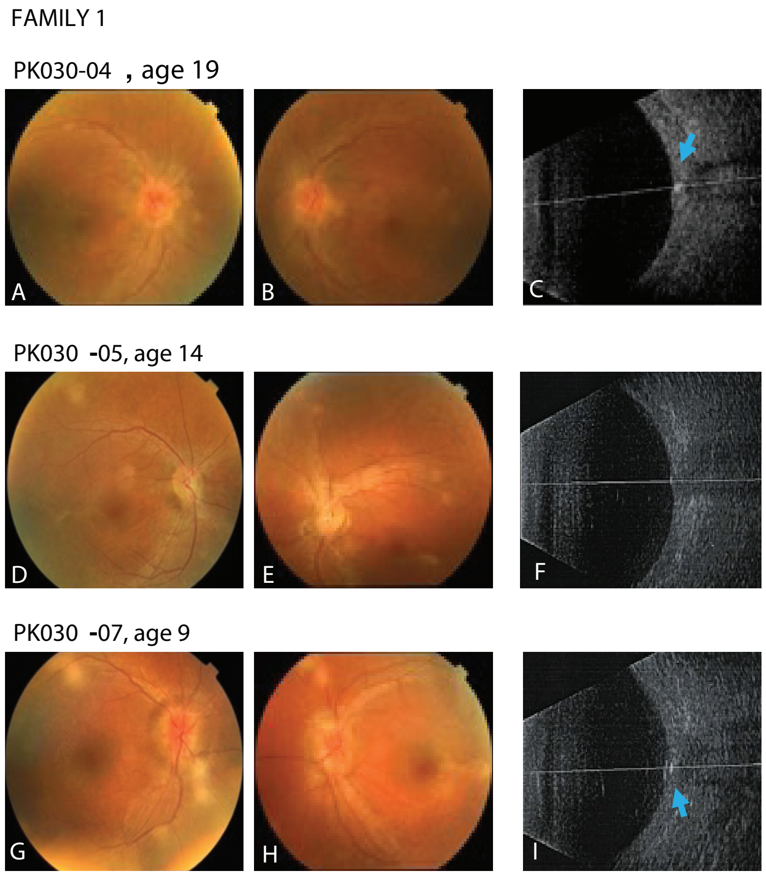


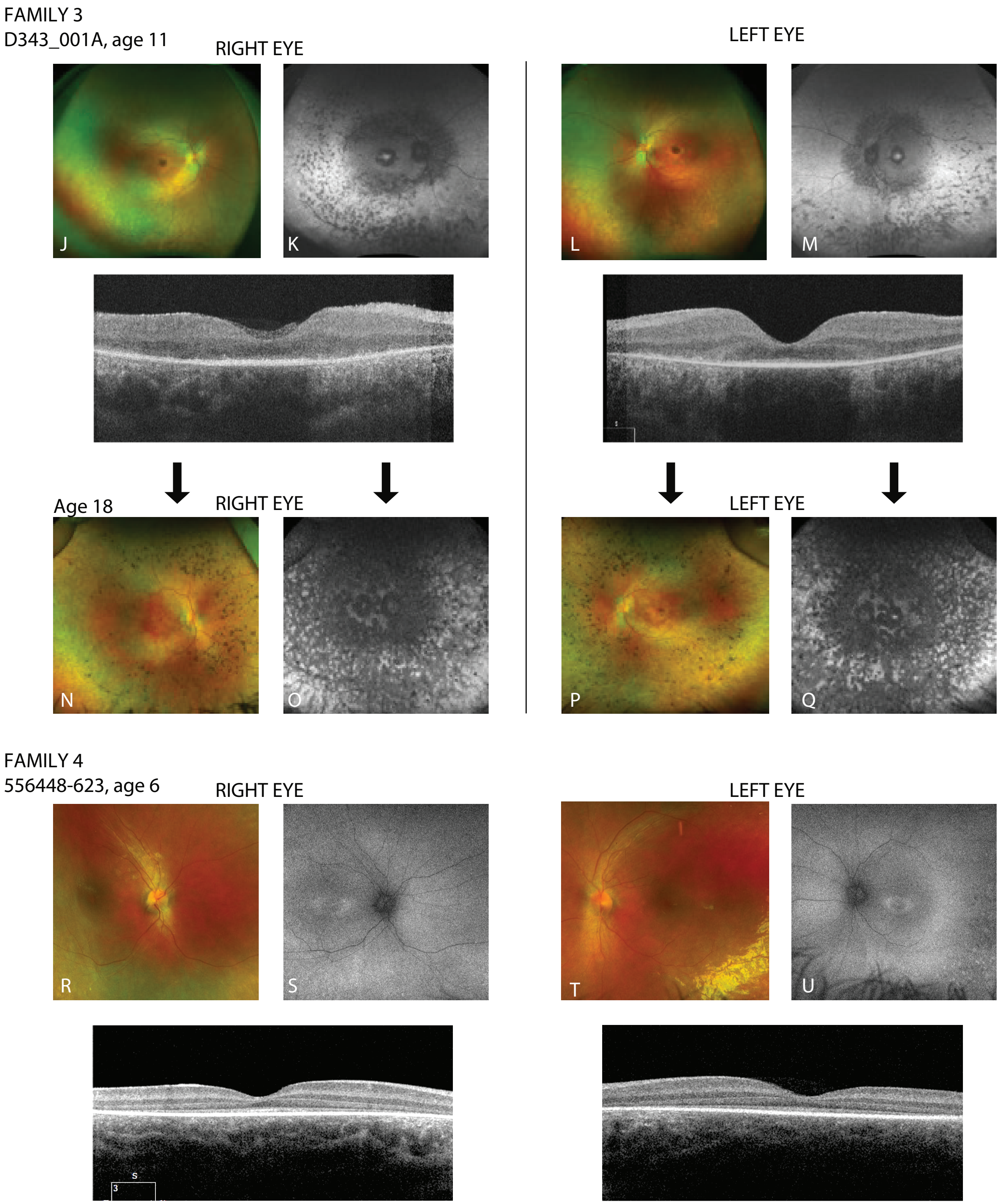


**eFigure 1: Ocular features of *SLC6A6*-associated retinopathy.** (A-C) Patient PK030-04 (Family 1, 19 years old). Color fundus photography of the right (A) and left (B) eye showing attenuated retinal vasculature and blurred optic disc margins. (C) B-scan ultrasonography of the right eye showing calcified optic disc drusen (ODD) as a hyperechoic structure (cyan arrow). (D-F) Patient PK030-05 (Family 1, 14 years old). Color fundus photography of the right (D) and left (E) eye showing attenuated retinal vasculature and slightly blurred optic disc margins, especially on the nasal side. (F) B-scan ultrasonography did not detect calcified ODD. (G-I) Patient PK030-07 (Family 1, 9 years old). Color fundus photography of the right (G) and left (H) eye showing attenuation of the retinal vessels and optic disc elevation with blurred margins, and nodular appearance of the optic disc border. (I) B-scan ultrasonography of the right eye confirming the presence of calcified ODD (cyan arrow). (J, L) Wide-field color fundus photography, and (K, M) mid-wavelength fundus autofluorescence of the right and left eye of the proband (D343_001A) of Family 3 at age 11 years, showing bull's-eye maculopathy and widespread macular and peripheral RPE pathology. Respective OCT scans are also shown. (N, P) Wide-field color fundus photography and (O, Q) mid-wavelength fundus autofluorescence of the right and left eye of the proband at age 18 (7 years later), showing progression of retinal degeneration. (R, T) Color fundus photography, and (S, U) fundus autofluorescence of the right and left eye of the proband (556448-623) of Family 4 at age 6, with relative OCT scans.

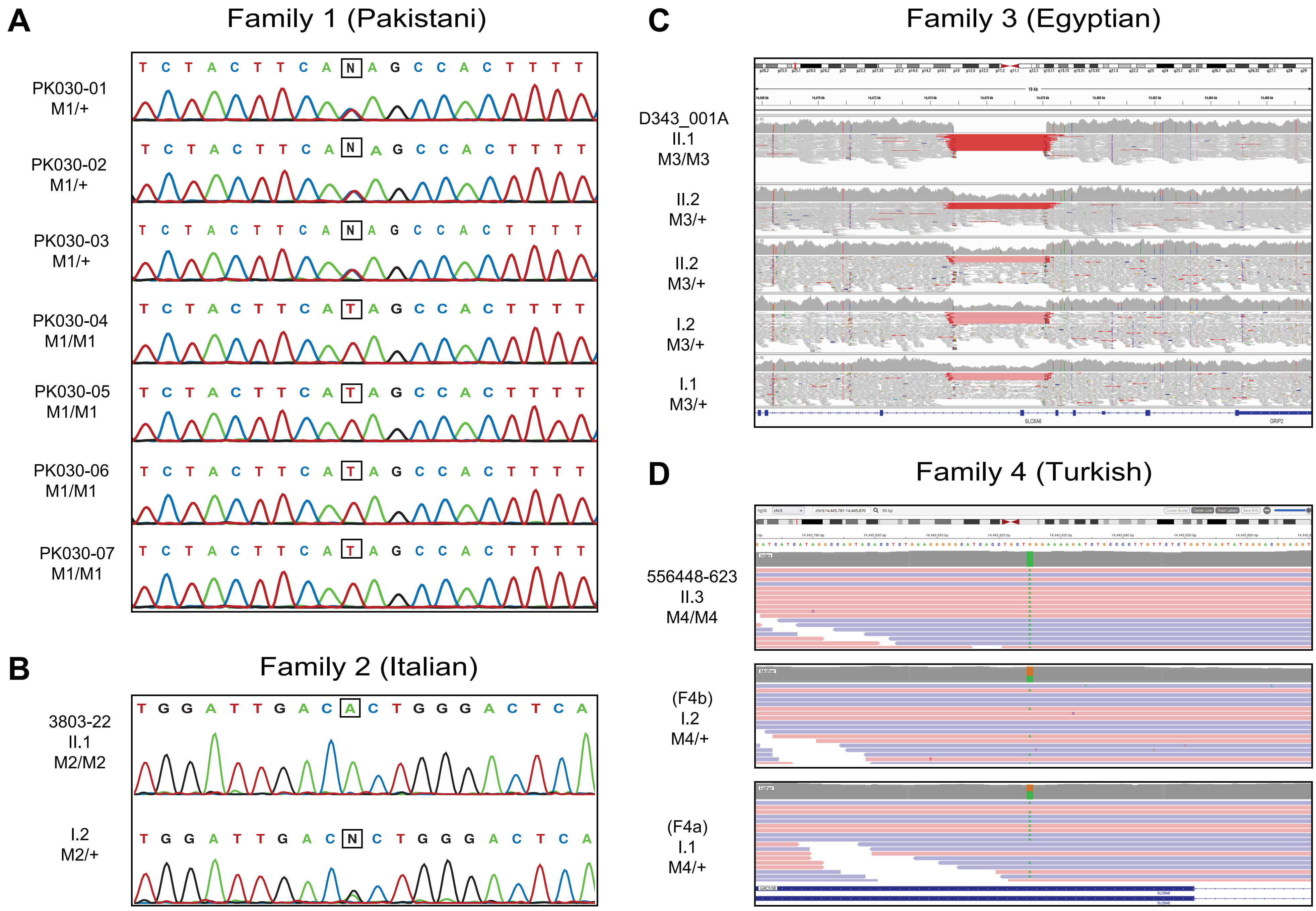


**eFigure 2: Additional data on genotyping and co-segregation analyses.** (A, B) Sanger sequencing of DNA from members from Families 1 and 2. (C, D) IGV visualization of genome data from members of Families 3 and 4.

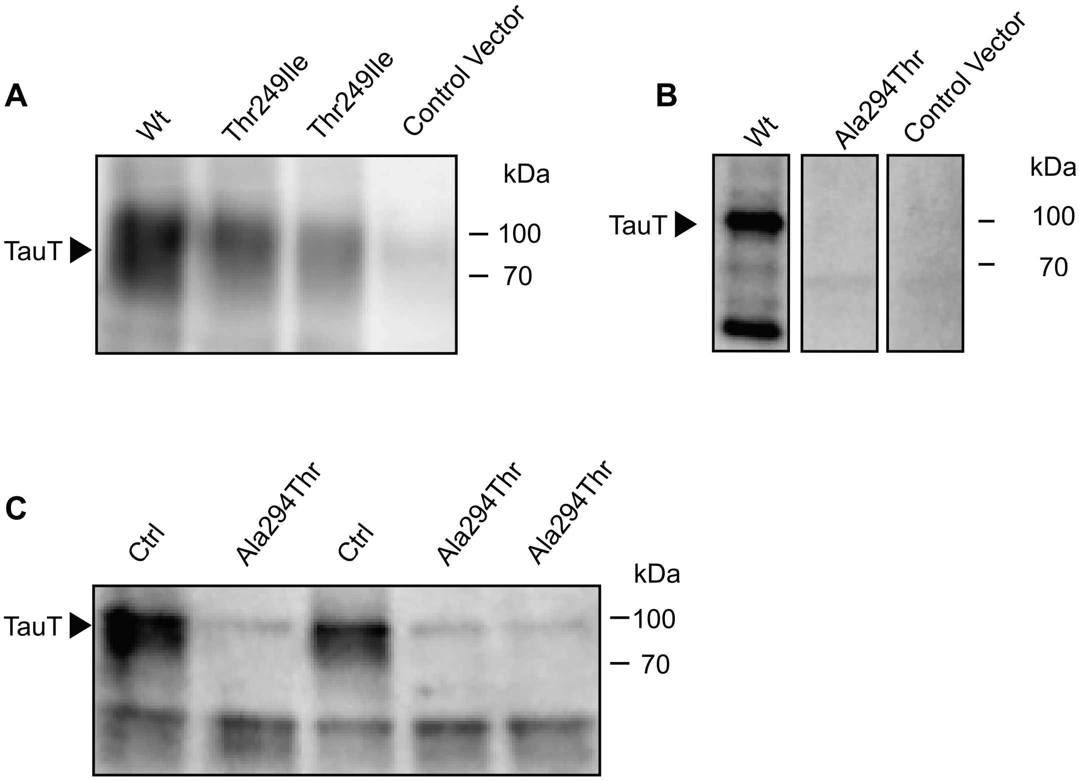


**eFigure 3. Representative surface biotinylation immunoblots from HEK-MSR and fibroblasts expressing p.(Thr249Ile) or p.(Ala294Thr) variants.**

Surface biotinylation immunoblots from HEK-MSR cells expressing wildtype or (A) Thr249Ile TauT or (B) Ala294Thr TauT. Panel C is a surface immunoblot from patient-derived fibroblasts from affected p.(Ala294Thr) or unaffected (Ctrl) individuals. Each panel in (B) is from the same blot, lanes rearranged for consistency.

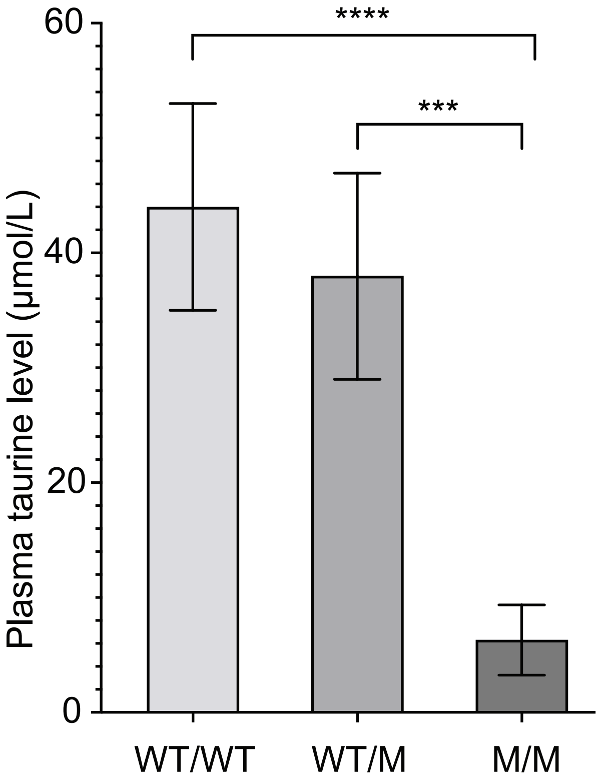


**eFigure 4. Plasma taurine levels in patients and controls**. The bar graph represents the mean of plasma taurine levels of control individuals (wild type, n=40), healthy heterozygous relatives of Family 1 and Family 4 (n=5) and of the patients (n=7). For statistical significance, an unpaired t-test with Welch's correction was performed to calculate the P value. ****P<.0001 for WT/WT vs M/M and ***P<.001 for WT/M vs M/M

## eTable 1: Demographic and clinical characteristics of all 7 patients carrying biallelic variants in *SLC6A6*

| | | Family 1 | | | | Family 2 | Family 3 | Family 4 |
|---|---|---|---|---|---|---|---|---|
| Demographics | Patient ID | PK030-04 | PK030-05 | PK030-06 | PK030-07 | 3803-22 | D343_001 | 556448-623 |
| | Variant info | NM_003043.5:c.746C>T; p.(Thr249Ile) | NM_003043.5:c.746C>T; p.(Thr249Ile) | NM_003043.5:c.746C>T; p.(Thr249Ile) | NM_003043.5:c.746C>T; p.(Thr249Ile) | NM_003043.5:c.880G>A; p.(Ala294Thr) | NM_003043.6:c.1210-2389_1348-331del; p.(Phe404_Glu449del) | NM_003043.6:c.338G>A; p.(Trp113Ter) |
| | Country of Recruitment | Pakistan | Pakistan | Pakistan | Pakistan | Italy | USA | France |
| | Ethnic origin | Pakistani | Pakistani | Pakistani | Pakistani | Italian | Egyptian | Turkish |
| | Sex | Male | Female | Female | Male | Female | Female | Female |
| | Age at examination | 19 yr | 14 yr | 12 yr | 9 yr | 5-9 yr | 11-18 yr | 1-7 yr |
| Ocular findings | Best-corrected visual acuity | OU: hand motions | OU: hand motions | OU: hand motions | OU: hand motions | OU: 20/63 (at age 5); OU worse than 20/200 (at age 9) | OD worse than 20/800, OS: 20/800 (at age 11).<br>OU: hand motions (at age 18) | OU: poor vision (at age 1) |
| | Refraction / axial length | OU: hyperopic | OU: hyperopic | OU: hyperopic | OU: hyperopic | OD: +2 sphere +2 cylinder axis 100°, OS: +2 sphere +1.50 cylinder axis 90° (at age 9, cycloplegia) | OD: +5 sphere, OS: +5.75 sphere +0.25 cylinder axis 64° (at age 11, cycloplegia).<br>Axial length: 20.01 mm (OD), 19.93 mm (OS) (at age 18) | OU: hyperopic (at age 1) |
| | Eye movements | Nystagmus | Nystagmus | Nystagmus | Nystagmus | Alternating fixation, smooth pursuit impairment, hypometric saccades (at age 5). Horizontal nystagmus with a torsional component, variable alternating exotropia (at age 9) | Variable intermittent exotropia, limited supraduction in abduction, moderate-frequency, moderate-amplitude horizontal nystagmus (at age 11). Unchanged at age 18 | Nystagmus (at age 1) |
| | Anterior segment | OU: normal | OU: normal | OU: normal | OU: normal | OU: normal (at age 9) | OU: embryonic nuclear and sutural cataract, non-visually significant (at age 11). Unchanged at age 18 | NA |
| | Fundus | OU: attenuated retinal vessels, RPE defects and pigment clumps in the peripheral retina, blurred optic disc margins (calcified ODD confirmed by B-scan ultrasonography) | OU: attenuated retinal vessels, RPE defects in the peripheral retina, blurred optic disc margins | OU: attenuated retinal vessels, RPE defects in the peripheral retina, blurred optic disc margins | OU: attenuated retinal vessels, RPE defects and pigment clumps in the peripheral retina, blurred optic disc margins (calcified ODD confirmed by B-scan ultrasonography) | OU: normal (at age 5) | OU: mildly attenuated retinal vessels, macular RPE atrophy in a bull's-eye configuration with pigment deposition in the macula, fine RPE mottling in the peripheral retina, mildly pale optic discs with ODD confirmed by enhanced depth imaging OCT and B-scan ultrasonography (at age 11).<br>OU: progression of macular atrophy and clumped pigmentary retinopathy at age 18 | OU: thin retinal vessels, no pigmentary changes (at age 1) |
| | OCT | NA | OU: central island of relatively preserved ONL abnormally declining in thickness with eccentricity, ELM/EZ bands not well delineated and limited to a central island. CST: 189 µm (OD), 191 µm (OS) | OU: central island of relatively preserved ONL abnormally declining in thickness with eccentricity, ELM/EZ bands not well delineated and limited to a central island. CST: 194 µm (OD), 191 µm (OS) | NA | OU: central island of relatively preserved ONL and RPE, abnormal ONL thinning with eccentricity (at age 9) | OU: central island of relatively preserved ONL and RPE, abnormal decline in ONL thickness with eccentricity past the parafovea, ELM/EZ bands not well delineated and limited to a central island. CST: 256 µm (OD), 226 µm (OS) (at age 11) | OU: central island of relatively preserved ONL, abnormal decline in ONL thickness with eccentricity (at age 6) |
| | Full-field electroretinography | OU: severely depressed scotopic & photopic responses | OU: severely depressed scotopic & photopic responses | OU: severely depressed scotopic & photopic responses | OU: severely depressed scotopic & photopic responses | OU: depressed scotopic and photopic response (at age 9) | OU: extinguished scotopic and photopic responses (at age 11) | OU: severely reduced scotopic and photopic responses (at age 4) |
| Non ocular findings | Electrocardiography[#] | Normal SR, short PR interval (110 ms), LVH criteria, T-wave inversion (V4–V6, DII, DIII, aVF) | Short PR interval (110 ms) | Short PR interval (110 ms) | Short PR interval (110 ms) | Tachycardic SR, short PR interval (80 ms), slight right conduction delay | Normal SR (60–99 bpm), early transition in V1 with R>S | Normal sinus rhythm (99 bpm), QTc: 413 ms (at age 7) |
| | Echocardiography | Normal EF and FS | Normal EF and FS | Normal EF and FS | Normal EF and FS | Normal; preserved LV size and function (LVIDd = 42 mm; EDV = 59 mL; EF = 60%) | EF: 56%; no dilation or hypertrophy of cardiac chambers | Normal; EF: 65% (at age 7) |
| | Neurological and other | None | None | None | None | Mild to moderate intellectual disability (IQ 62), developmental motor coordination disorder, brain MRI, EEG and brainstem auditory evoked potentials within normal limits (at age 5) | Mild intellectual disabilities. Pectus carinatum. Deep venous thrombosis (at age 15) treated with apixaban, followed by thrombectomy and stent placement: she was found to be heterozygous for the Factor V Leiden mutation (F5:NM_000130.4:c.1601G>A: p.Arg534Gln, also commonly known as R506Q). Normal hearing | Born at 38 weeks following an unremarkable pregnancy, with measurements within normal ranges. Facial dysmorphic features and motor dysfunction (at age 7 months). Asthma. EEG and brain MRI normal (at age 2). Liver and renal imaging unremarkable (at age 7) |
| | Plasma taurine level (normal range = 36-52 µmol/L)* | 10 | 8 | 10 | 4 | 6 | 5 | 1 |

bpm = beats per minute, CME = cystoid macular edema, CST = central subfield thickness, EDV = end-diastolic volume, EEG = electroencephalography, EF = ejection fraction, ELM = external limiting membrane, EZ = ellipsoid zone, FS = fractional shortening, LV = left ventricular, LVIDd = left ventricular internal diameter in diastole, MRI = magnetic resonance imaging, ms = milliseconds, NA = not available, ODD = optic disc drusen, OD = right eye, ONL = outer nuclear layer, OS = left eye, OU = both eyes, RPE = retinal pigment epithelium, SR = sinus rhythm. [#] normal PR interval = 120-200 ms. * quantification was done at the age of last examination.

**eTable 2.** Results from in silico tools assessing all variants reported in this study

| ID | Type of variant | Gene | Method | cDNA position | Protein position | Number of patients | ACMG | ACMG criteria | gnomAD v2.1.1 | gnomAD v4.1.0 | MutScore | AlphaMissense | VEST4 | REVEL |
|---|---|---|---|---|---|---|---|---|---|---|---|---|---|---|
| PK030-04 | Missense | SLC6A6 | WES | NM_003043.5:c.746C>T | NP_003034.2:p.(Thr249Ile) | 4 | P | PS3_strong, PM2_mod, PP1_strong, and PP3_mod | . | . | 0.792 | 0.946 | 0.978 | 0.905 |
| 3803-22 | Missense | SLC6A6 | WES | NM_003043.5:c.880G>A | NP_003034.2:p.(Ala294Thr) | 1 | LP | PS3_strong, PM2_mod, and PP3_mod | 4.00E-06 | 2.50E-06 | 0.81 | 0.889 | 0.961 | 0.886 |
| D343_001A | Deletion | SLC6A6 | WGS | NM_003043.6:c.1210-2389_1348-331del | NP_003034.2:p.(Phe404_Glu449del) | 1 | LP | PVS1_strong and PM2_mod | . | . | NA | NA | NA | NA |
| 556448-623 | Stop gain | SLC6A6 | WGS | NM_003043.6:c.338G>A | NP_003034.2:p.(Trp113Ter) | 1 | LP | PVS1_strong and PM2_mod | . | . | NA | NA | NA | NA |

WES - Whole Exom Sequencing; WGS - Whole Genome Sequencing
P - pathogenic; LP - likely pathogenic
. - absent
NA - not applicable
