# Supplementary material for: Early-Onset Retinopathy in Patients With Variants in SLC6A6 Leading to Impaired Taurine Transport
Source: JAMA Ophthalmol. 2025 Dec 4;144(1):70–8. doi: 10.1001/jamaophthalmol.2025.4875 (PMC12679424; doi:10.1001/jamaophthalmol.2025.4875)
Supplement: Supplement 2. — Data Sharing Statement [file jamaophthalmol-e254875-s002.pdf]

Data Sharing Statement

Ullah. Early-Onset Retinopathy in Patients With Variants in SLC6A6 Leading to Impaired Taurine Transport. *JAMA Ophthalmol*. Published December 04, 2025. doi:10.1001/jamaophthalmol.2025.4875

Data
Data available: No
